# The role of the giacomini vein in preoperative mapping of lower limb varicose veins

**DOI:** 10.1590/1677-5449.202400582

**Published:** 2024-11-29

**Authors:** Carlos Alberto Engelhorn, Ana Luiza Dias Valiente Engelhorn, Elisa da Silva de Oliveira, Julia Marques de Macedo, Leticia Bressan Anizelli, Maria Luiza Oliveira de Mendonça

**Affiliations:** 1 Pontifícia Universidade Católica do Paraná – PUCPR, Curitiba, PR, Brasil.

**Keywords:** ultrasonography, Doppler, venous insufficiency, varicose veins, preoperative period

## Abstract

**Background:**

The Giacomini vein (GV) can transfer reflux from perineal veins, tributary veins, and perforators of the thigh to the small saphenous vein (SSV). Vascular ultrasound with Doppler (VUD) is the preferred method for detecting reflux in specific veins such as the GV.

**Objective:**

To identify GV depth and diameter, reflux in the GV, and presence of reflux in the SSV caused by the GV.

**Methods:**

A cross-sectional, retrospective study was conducted in women undergoing lower limb venous mapping for varicose vein surgery. The following parameters were analyzed in GVs in which reflux was detected: segmental or diffuse reflux pattern; GV diameter and depth; and reflux in the SSV caused by the GV.

**Results:**

340 of the 2368 women evaluated were included in the study because they had a GV, totaling 511 veins analyzed, 150 (29.4%) of which had reflux. The diameters of the 150 GVs with reflux ranged from 1.5 to 7.8 mm and their depth varied from 4 to 25 mm. Most GVs with reflux (91.3%) had a segmental reflux pattern. The majority (66%) of refluxing GVs drained reflux into the popliteal vein through the saphenopopliteal junction, while reflux was transferred to the SSV in 34 veins (22.7%), and was drained by a tributary vein in the thigh in 15 veins (11.3%).

**Conclusions:**

Approximately one-third of the studied GVs had reflux, mostly segmental, mean caliber was 2.7 mm, and mean depth was 11 mm. Reflux in the SSV originating from the GV was detected in 22% of the evaluated veins.

## INTRODUCTION

The Giacomini vein (GV) is a tributary vein of the great saphenous vein (GSV) or the posterior accessory saphenous vein that ascends obliquely in the posterior thigh, with both subfascial and subcutaneous segments, and is, therefore, an intersaphenous vein. The GV may originate from the small saphenous vein (SSV) or from its cranial extension.^1^

This intersaphenous vein originates superficially in the popliteal fossa, arising from the segment between the pre-terminal and terminal valves of the SSV and initially ascends between the semimembranosus and biceps femoral muscles and then within the sulcus between the biceps femoral muscle and the semitendinosus muscle, alongside the posterior femoral cutaneous nerve. Its valves are configured to direct blood from the SSV to the GSV, preventing distal reflux, and can be found in both the subfascial portion and the subcutaneous segment, close to the junction with the GSV.^2^

Valve incompetence in the GV can be responsible for emergence of varicose veins in the posterior thigh or in the topography of the SSV and must be identified in preoperative assessments to ensure better results of surgical treatment.

Vascular ultrasonography with Doppler (VUSD) has been used for more than twenty years for detecting and assessing reflux of blood in veins of the lower limbs (LL), primarily using color flow mapping (to identify retrograde flow) and spectral Doppler (reflux time). It is thus possible to precisely identify the distribution and extent of venous reflux. This examination has become the method of choice for assessment of the peripheral venous system.^3^

In order to detect sources of reflux and their repercussions for the superficial vein system and chronic venous disease, it is necessary to identify incompetent venous segments that cause varicose veins, such as a GV with reflux.

The GV can be a source of reflux into the SSV, giving rise to varicose veins in the posterior leg, and may constitute indications for surgery, which should also include the GV and can be performed with endovenous procedures. It is therefore important to detect reflux and identify the specific reflux pattern present in the GV.

The objectives of this study were to identify presence of GVs; their depth and diameter, reflux in the GV, and any possibility of reflux in the SSV caused by the GV.

## METHODS

A cross-sectional, retrospective, observational study was conducted of data from 2,368 women referred to the Angiolab vascular laboratory (Curitiba, Paraná, Brazil), for venous mapping. Inclusion criteria were age greater than 18 years and primary varicose veins in the LL. Patients with a prior history of surgery for lower limb varicose veins were excluded.

A sample size calculation was conducted for a 0.05 margin of error and 0.95 confidence level and considering a mean prevalence in the literature of 29%, indicating a minimum sample size of 317 GVs.

### Ultrasonographic assessment

The ultrasonographic assessment was conducted using Siemens-Acuson Antares® and X 700® ultrasound machines (Issaquah®, United States), with 5 MHz transducers to assess the deep vein system and rule out deep venous thrombosis, with the patient lying down, and 7 MHz transducers to assess the superficial vein system, with the patient standing upright.

The GSV and SSV were examined and presence of GVs identified with the patient standing upright, acquiring anatomic images of the veins in transverse and longitudinal views with the ultrasound equipament in B-mode.

**Figure 1:**
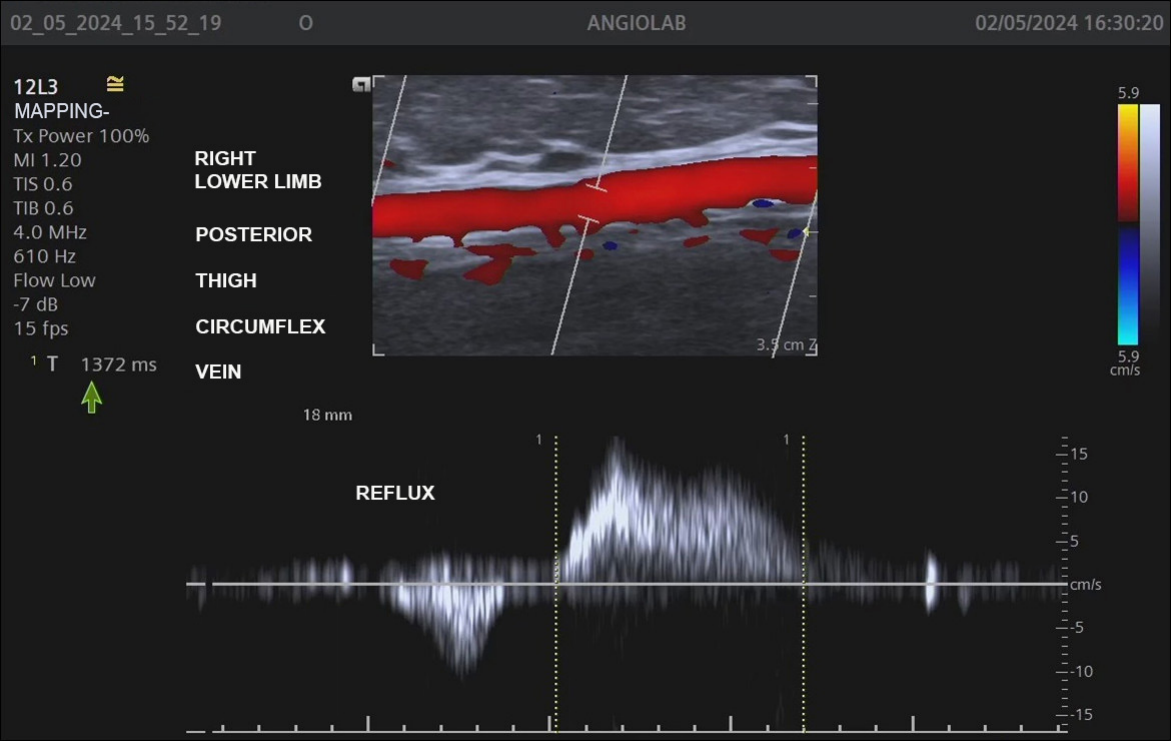
Measurement of reflux time (1.3 sec) in the Giacomini vein.

With the help of color flow mapping and spectral Doppler, valve competence was assessed by applying manual muscle compression, distal of the transducer, in order to provoke and detect reflux in the saphenous veins and GV, when present. Reflux in these veins was defined as present if there was retrograde flow with a duration exceeding 0.5s^4^ (Figure 1).

**Figure 2:**
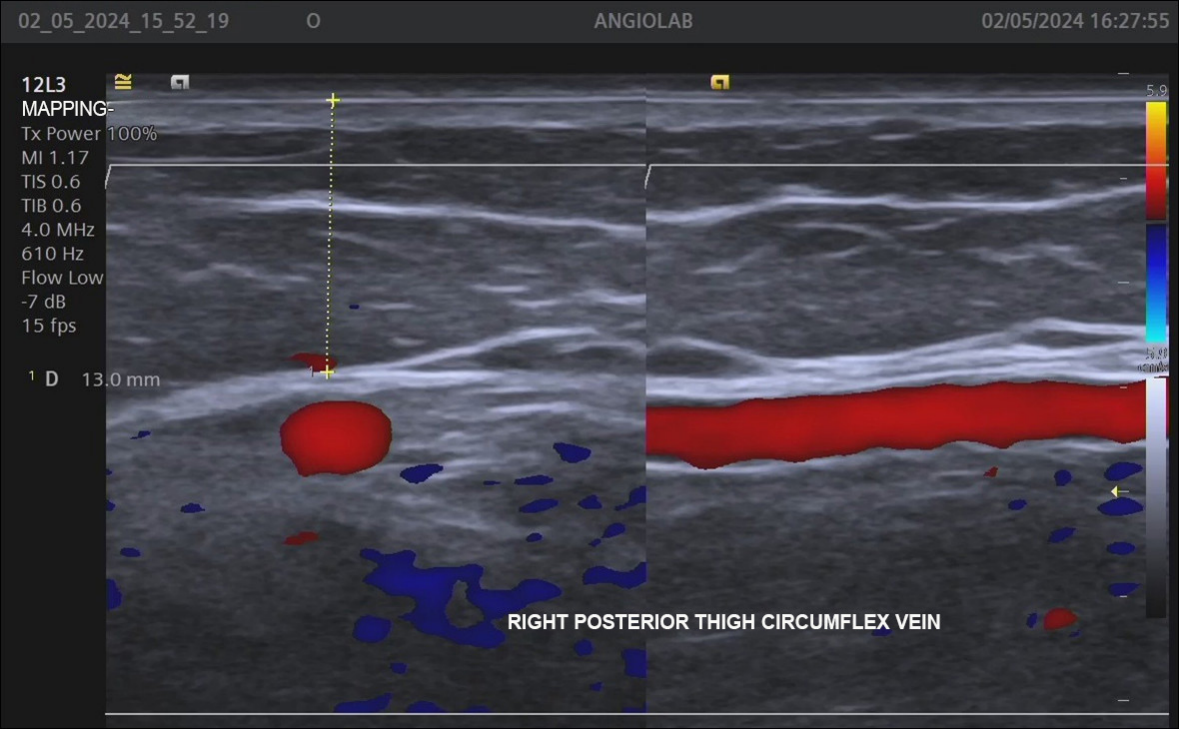
Measurement of the depth (13 mm) of the Giacomini vein, in the muscular fascia in relation to the skin.

The GV-specific assessment considered the following parameters: reflux absent, segmental, or diffuse; presence of reflux in the SSV caused by the GV; and diameter, depth, and height (distance above the sole of the foot) of communication with the GSV and the SSV (for GVs with reflux only) (Figure 2).

For statistical analysis, quantitative variables were expressed as mean, standard deviation, median, and range. For categorical variables, frequency and percentage were calculated. Data were organized in an Excel^®^ spreadsheet and analyzed with IBM SPSS Statistics v.28.0 (Armonk: IBM Corp).

The study was approved by the Research Ethics Committee at the Pontifícia Universidade Católica do Paraná (PUCPR), under decision number 3.987.576.

## RESULTS

A total of 340 out of the 2,368 women assessed (14.3%) had GVs and were included in the study, totaling 511 veins analyzed, with a similar distribution between limbs. Half of the sample had bilateral GVs. Of the 511 GVs analyzed, 150 (29.4%) had reflux. The diameter of these 150 GVs with reflux ranged from 1.5 to 7.8 mm (mean of 2.7 mm); mean depth was 11.5 mm, varying from 4 to 25 mm; and the level of the connections with the GSV and with the SSV ranged from 60 to 82 cm (mean of 71 cm) and from 40 to 60.5 cm (mean of 50.5 cm), respectively (Table 1).

**Table 1:** Diameter, depth, and connections of Giacomini veins.

**Variable**	**n**	**Mean**	**Standard deviation**	**Median**	**Minimum**	**Maximum**	**95% confidence interval**
Diameter	150	2.7	0.8	2.6	1.5	7.8	2.57-2.82
Depth	150	11.5	3.5	11.0	4.0	25.0	10.94-12.06
Height, GSV	138	71.0	4.7	71.0	60.0	82.0	70.22-7.78
Height, SSV	148	50.4	4.3	50.0	40.0	60.5	49.71-51.09

GSV: great saphenous vein; SSV: small saphenous vein.

Among GVs with reflux (29.4% of the sample), the great majority (91.3%) had segmental reflux and just 8.7% of the veins had diffuse reflux. The origin of reflux in 98% (147) of the veins with segmental reflux was a tributary vein on the posterior aspect of the thigh (Table 2).

**Table 2:** Patterns and sources of reflux in Giacomini veins.

**Variable**	**Total**	**Classification**	**N**	**%**
Diffuse reflux	150	0	137	91.3
		1	13	8.7
Segmental reflux	150	0	13	8.7
		1	137	91.3
CAUSE - Tributary	150	0	3	2.0
		1	147	98.0
CAUSE – Perforator	150	0	147	98.0
		1	3	2.0

**Figure 3:**
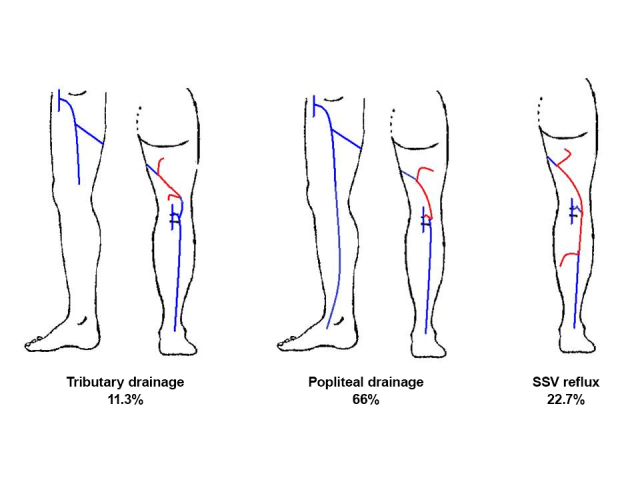
Diagram illustrating the drainage possibilities of reflux from the Giacomini vein. SSV = small saphenous vein.

With relation to drainage of the reflux in the 150 incompetent veins, the majority (66%) drained reflux to the popliteal vein via the saphenopopliteal junction (SPJ); in 34 veins (22.7%), reflux was transferred to the SSV, and in 15 veins (11.3%) it was drained by a tributary vein in the thigh (Figure 3).

**Figure 4:**
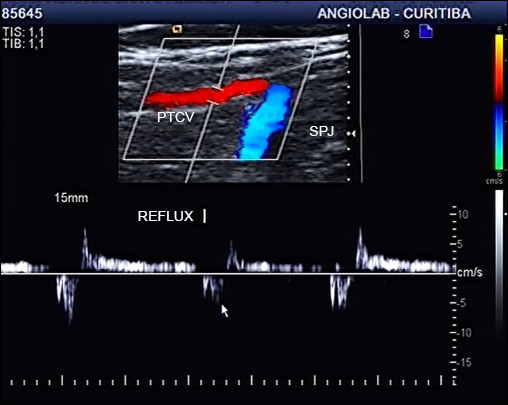
Ultrasound image of Giacomini vein with reflux. PTCV = Posterior thigh circumflex vein (Giacomimni Vein); SPJ = saphenopopliteal junction.

Figures 4 [Fig gf0500] to 7 illustrate examples of reflux in the GV draining into the SPJ without compromising the SSV and of reflux in the SSV caused by an incompetent GV.

**Figure 5 gf0500:**
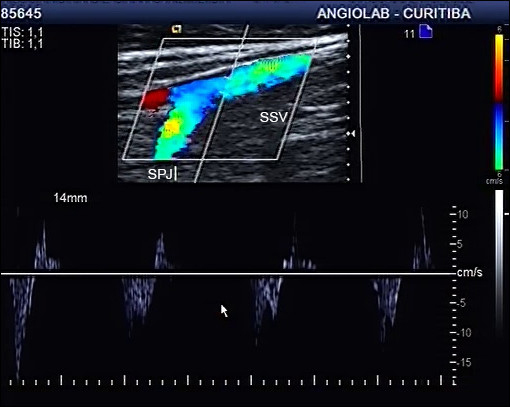
Ultrasound image of the small saphenous vein (same case as in Figure 4) without reflux, demonstrating that reflux in the Giacomini vein is drained via the saphenopopliteal junction. SSV = Small saphenous vein; SPJ = saphenopopliteal junction.

**Figure 6:**
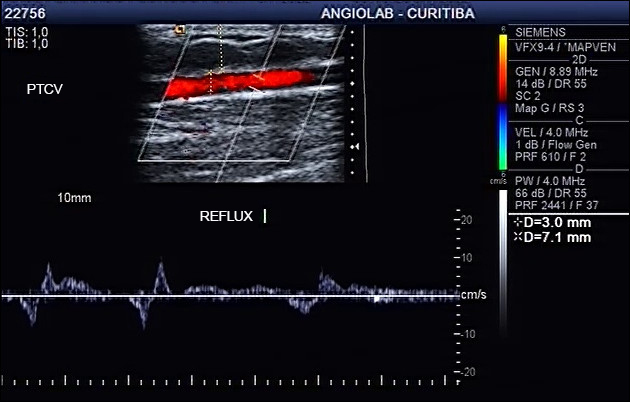
Ultrasound image of the Giacomini vein with reflux, with caliber of 3 mm and depth of 7.1 mm. PTCV = Posterior thigh circumflex vein (Giacomini Vein).

**Figure 7:**
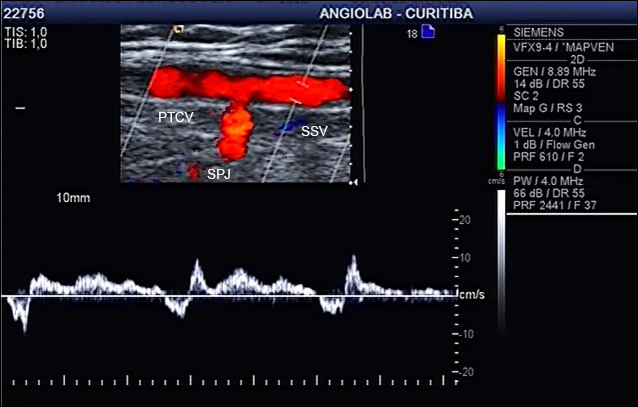
Ultrasound image of the small saphenous vein (same case as in Figure 6) with reflux, demonstrating that reflux in the Giacomini vein is transferred to the small saphenous vein. PTCV = Posterior thigh circumflex vein; SSV = small saphenous vein; SPJ = saphenopopliteal junction.

## DISCUSSION

The definition of a GV is a little controversial in the literature. Carlo Giacomini’s original description, from 1873, lists eight different types. Type 1, the definition adopted in our study, is the most common (52.9%) and is described as an anastomotic branch between the SSV (terminating at the popliteal vein) and the GSV.^5^

In 2001, the International Union of Phlebology, with the support of the Federative International Committee on Anatomical Terminology, established new terminology for the superficial veins of the lower limbs, in which the intersaphenous vein corresponds to the Giacomini vein. Along the same lines, in 2002, the International Interdisciplinary Consensus Committee on Venous Anatomical Terminology stated that when the cranial continuation of the SSV communicates with the GSV (via the posterior thigh circumflex vein), it should be called the GV.^6,7^

The prevalence of GVs varies from 2.5% in a phlebographic study,^8^ through 2 to 86% in ultrasound assessments,^9,10^ and even as high as 95% in cadaveric studies.^11-13^

In our study, the prevalence observed with ultrasonography was 14.5% of a specific population of women referred for venous mapping for varicose veins surgery.

With relation to the caliber of the 150 GVs with reflux assessed in our study, diameters ranged from 1.5 to 7.8 mm (mean of 2.7 mm) and mean depth was 11.5 mm, varying from 4 to 25 mm. Delis et al. reported a similar mean caliber (2.68 mm) to our study, with a range of 0.2 to 7.7 mm.^14^

Considering that the GV has both subfascial and subcutaneous segments, we believe that the depth of a GV with reflux is relevant information in the context of possible endovascular treatment. This information is not available elsewhere in the literature.

Six different SSV reflux patterns are described, including perijunctional, proximal, segmental, multisegmental (with and without SPJ involvement), and distal reflux.^15,16^

Among these patterns, the perijunctional type (reflux in the SSV below the SPJ) is directly related to the GV. In this pattern, the incompetent GV transfers reflux to the SSV below the SPJ, making it incompetent and potentially originating varicose veins in the leg. In these cases, failure to treat the GV may lead to relapse in the future.

In the literature, rates of reflux in the GV detected by VUSD vary from 2 to 19%.^17,18^ Our study identified reflux in 29.4% of GVs, which was segmental in the great majority (91.3%) and was diffuse in just 8.7%. In the majority of cases (66%), reflux is drained by the SPJ, without causing reflux in the SSV. The higher incidence of reflux in our study may be related to the fact that GV assessment was performed routinely in our vascular laboratory.

Among the incompetent GVs, 22.7% transferred reflux to the SSV, constituting the perijunctional reflux pattern. Veltman et al. observed 10% perijunctional reflux with VUSD in 1,142 lower limbs, caused by Giacomini veins or cranial continuation of the SSV.^19^

Considering the 29% incidence of reflux in the GV vein and the possibility of a perijunctional reflux pattern in 23% of SSVs, we believe that preoperative mapping of lower limbs varicose veins should routinely include proactive screening for the GV and assessment of GVs when found.

In cases with GV reflux, the caliber and height of the connections with the GSV and SSV should be measured. In our study they had mean height (above the sole of the foot) of 71 cm for the GSV and 50.5 cm for the SSV.

In conclusion, the incidence of GV presence in this study was 14%. One third of these veins had reflux, which was segmental in the majority of cases. In 23% of GV, reflux was transferred to the SSV, which makes assessment relevant during preoperative mapping of varicose veins and for planning surgery.
