# Supplementary material for: The role of the giacomini vein in preoperative mapping of lower limb varicose veins
Source: J Vasc Bras. 2024 Nov 29;23:e20240058. doi: 10.1590/1677-5449.202400582 (PMC11614100; doi:10.1590/1677-5449.202400582)
Supplement: Translation

# Translation: O papel da veia de Giacomini no mapeamento pré-operatório das varizes dos membros inferiores

**DOI:** 10.1590/1677-5449.202400581

**Authors:** Carlos Alberto Engelhorn, Ana Luiza Dias Valiente Engelhorn, Elisa da Silva de Oliveira, Julia Marques de Macedo, Leticia Bressan Anizelli, Maria Luiza Oliveira de Mendonça

**Affiliations:** 1 Pontifícia Universidade Católica do Paraná – PUCPR, Curitiba, PR, Brasil.

**Keywords:** ultrassonografia Doppler, insuficiência venosa, varizes, período pré-operatório

## Abstract

**Contexto:**

A veia de Giacomini (VG) pode transferir refluxo de veias perineais, veias tributárias e veias perfurantes da coxa para a veia safena parva (VSP). A ultrassonografia vascular com Doppler (USVD) é o exame de escolha para detecção do refluxo em veias específicas como a VG.

**Objetivos:**

Identificar a profundidade, o diâmetro e o refluxo na VG e a presença de refluxo na VSP causado pela VG.

**Métodos:**

Estudo transversal e retrospectivo, em mulheres que realizaram mapeamento venoso para cirurgia de varizes de membros inferiores. Nas VGs com refluxo, foram considerados os seguintes parâmetros: refluxo segmentar ou difuso; diâmetro, profundidade; e refluxo na VSP causado pela VG.

**Resultados:**

Das 2.368 mulheres avaliadas, 340 foram incluídas no estudo por apresentarem VG, totalizando 511 veias analisadas, sendo 150 (29,4%) veias com refluxo. Nas 150 VGs com refluxo, o diâmetro variou entre 1,5 e 7,8 mm e a profundidade, entre 4 e 25 mm. O padrão de refluxo na maioria das VGs (91,3%) foi do tipo segmentar. Em relação à drenagem do refluxo das VGs, a maioria (66%) drenou o refluxo para a veia poplítea através da junção safenopoplítea; em 34 veias (22,7%), o refluxo foi transferido para a VSP e, em 15 veias (11,3%), foi escoado por veia tributária na coxa.

**Conclusões:**

Aproximadamente um terço das VGs estudadas apresentou refluxo, majoritariamente segmentar, com calibre médio de 2,7 mm e profundidade média de 11 mm. Refluxo na VSP originado da VG foi detectado em 22% das veias avaliadas.

## INTRODUÇÃO

A veia de Giacomini (VG) é uma veia tributária da veia safena magna (VSM) ou da veia safena acessória posterior (VSAP) que ascende obliquamente na parte posterior da coxa, apresentando segmentos subfascial e subcutâneo, sendo, portanto, uma veia intersafênica. Essa veia pode se originar na veia safena parva (VSP) ou em sua extensão cranial^1^ .

Essa veia intersafênica origina-se superficialmente na fossa poplítea, no segmento entre as válvulas pré-terminal e terminal da VSP, ascendendo inicialmente entre os músculos semimembranoso e bíceps femoral e então no sulco entre o músculo bíceps femoral e músculo semitendíneo, junto com o nervo cutâneo femoral posterior. Suas válvulas estão arranjadas de modo a direcionar o sangue da VSP até a VSM, impedindo o refluxo distal, e podem ser encontradas tanto na porção subfascial como na subcutânea próximo à junção com a VSM^2^ .

A insuficiência valvular da VG pode ser responsável pelo aparecimento de veias varicosas na região posterior de coxa ou no território da VSP e precisa ser identificada na avaliação pré-operatória para um melhor resultado do tratamento cirúrgico.

A ultrassonografia vascular com Doppler (USVD) tem sido utilizada há mais de duas décadas para detectar e avaliar refluxo sanguíneo em veias dos membros inferiores (MMII), principalmente com a utilização do mapeamento em cores do fluxo (identificação de fluxo retrógrado) e do Doppler espectral (tempo de refluxo). Assim, é possível identificar com precisão a distribuição e a extensão do refluxo venoso. Esse exame tornou-se o método de escolha para a avaliação venosa periférica^3^ .

Para detectar as fontes de refluxo e as repercussões no sistema venoso superficial e na doença venosa crônica, faz-se necessário identificar os segmentos venosos comprometidos, causadores de varizes, como a presença da VG com refluxo.

A VG pode ser fonte de refluxo para a VSP originando veias varicosas para a região posterior de perna, para as quais poderá haver indicação de cirurgia que deverá também abranger a VG, inclusive com procedimento endovenoso. Por isso, a importância de detectar refluxo e o padrão específico de refluxo na VG.

Os objetivos deste estudo foram identificar a presença, a profundidade, o diâmetro e o refluxo na VG e a possibilidade de refluxo na VSP causado pela VG.

## MÉTODOS

Foi realizado um estudo observacional transversal retrospectivo em 2.368 mulheres encaminhadas ao Angiolab Laboratório Vascular (Curitiba, Paraná), para realização de mapeamento venoso. Os critérios de inclusão foram idade superior a 18 anos e varizes primárias nos MMII. Foram excluídos homens e mulheres com cirurgia prévia de varizes de MMII.

O cálculo do tamanho da amostra realizado com margem de erro de 0,05 e nível de confiança de 0,95, considerando a prevalência média na literatura de 29%, indicou um tamanho amostral mínimo de 317 VGs.

### Avaliação ultrassonográfica

A avaliação ultrassonográfica foi realizada com equipamentos Siemens-Acuson Antares® e X 700® (Issaquah®, EUA), com transdutor de 5 MHz para avaliação do sistema venoso profundo e exclusão de trombose venosa profunda em decúbito e transdutor de 7 MHz para avaliação do sistema venoso superficial em ortostatismo.

O estudo da VSM e VSP e a identificação da VG foram realizados com o paciente em posição ortostática para a obtenção das imagens anatômicas das veias em cortes ultrassonográficos transversais e longitudinais em modo B.

Com o auxílio do mapeamento em cores do fluxo e Doppler espectral, avaliou-se o funcionamento valvular pela manobra de compressão muscular manual distal ao posicionamento do transdutor, a fim de produzir e detectar refluxo nas veias safenas e na VG, quando presente. Foi considerado refluxo nessas veias fluxo retrógrado com duração superior a 0,5 s^4^ (Figura 1).

**Figura 1:** Mensuração do tempo de refluxo (1,3 seg) na veia de Giacomini.

Para a avaliação específica da VG, foram considerados os seguintes parâmetros: refluxo ausente, segmentar ou difuso; presença de refluxo na VSP causado pela VG; e diâmetro, profundidade e altura (em relação à base do pé) da comunicação entre as VSM e VSP somente para VG com refluxo (Figura 2).

**Figura 2:** Mensuração da profundidade (13 mm) da veia de Giacomini, da fáscia muscular em relação à pele.

Na análise estatística, as variáveis quantitativas foram descritas por média, desvio-padrão, mediana, mínimo e máximo. Para variáveis categóricas, foram apresentados frequência e percentual. Os dados foram organizados em planilha Excel^®^ e analisados com o programa computacional IBM SPSS Statistics v.28.0 (Armonk: IBMCorp).

O estudo foi aprovado pelo Comitê de Ética em Pesquisa da Pontifícia Universidade Católica do Paraná (PUCPR), sob parecer número 3.987.576.

## RESULTADOS

Das 2.368 mulheres avaliadas, 340 (14,3%) apresentaram VG e foram incluídas no estudo, totalizando 511 veias analisadas, com distribuição semelhante entre as extremidades, sendo que metade das mulheres apresentou VG bilateralmente. Das 511 VGs analisadas, 150 (29,4%) apresentaram refluxo. Nessas 150 VGs com refluxo, o diâmetro variou entre 1,5 e 7,8 mm (média de 2,7 mm); a profundidade média foi de 11,5 mm, variando entre 4 e 25 mm; a altura das conexões entre a VSM e VSP variou entre 60 e 82 cm (média de 71 cm) e 40 e 60,5 cm (média de 50,5 cm), respectivamente (Tabela 1).

**Tabela 1:** Diâmetro, profundidade e conexões das veias de Giacomini.

**Variável**	**n**	**Média**	**Desvio-padrão**	**Mediana**	**Mínimo**	**Máximo**	**Intervalo de confiança de 95%**
Diâmetro	150	2,7	0,8	2,6	1,5	7,8	2,57-2,82
Profundidade	150	11,5	3,5	11,0	4,0	25,0	10,94-12,06
Altura VSM	138	71,0	4,7	71,0	60,0	82,0	70,22-7,78
Altura VSP	148	50,4	4,3	50,0	40,0	60,5	49,71-51,09

VSM: veia safena magna; VSP: veia safena parva.

Com relação à presença de refluxo (29,4%) na VG, a grande maioria (91,3%) apresentou refluxo segmentar, sendo somente 8,7% das veias com refluxo difuso. Em 98% (147) das veias com refluxo segmentar, o refluxo foi originado de veia tributária na face posterior de coxa (Tabela 2).

**Tabela 2:** Padrões e fontes de refluxo nas veias de Giacomini.

**Variável**	**Total**	**Classificação**	**N**	**%**
Refluxo difuso	150	0	137	91,3
		1	13	8,7
Refluxo segmentar	150	0	13	8,7
		1	137	91,3
CAUSA - Tributária	150	0	3	2,0
		1	147	98,0
CAUSA - Perfurante	150	0	147	98,0
		1	3	2,0

Em relação à drenagem do refluxo nas 150 veias insuficientes, a maioria (66%) drenou o refluxo para a veia poplítea através da junção safenopoplítea (JSP); em 34 veias (22,7%), o refluxo foi transferido para a VSP e, em 15 veias (11,3%), foi escoado por veia tributária na coxa (Figura 3).

**Figura 3:** Esquema exemplificando as possibilidades de drenagem do refluxo da veia de Giacomini. VSP = veia safena parva.

Exemplos de refluxo na VG drenando para a JSP sem comprometer a VSP e refluxo na VSP causado por VG insuficiente podem ser observados nas Figuras 4 [Fig gf05] a 7.

**Figura 4:** Imagem ultrassonográfica da veia de Giacomini com refluxo. VCPC = Veia Circunflexa Posterior de Coxa (Veia de Giacomini); JSP= Junção Safenopoplítea.

**Figura 5 gf05:** Imagem ultrassonográfica da veia safena parva sem refluxo (mesmo caso da Figura 4) sem refluxo, demonstrando que o refluxo da veia de Giacomini é drenado pela junção safenopoplítea. VSP= Veia Safena Parva; JSP = Junção Safenopoplítea.

**Figura 6:** Imagem ultrassonográfica da veia de Giacomini com refluxo, apresentando calibre de 3 mm e profundidade de 7,1 mm. VCPC = Veia Circunflexa Posterior de Coxa (Veia de Giacomini).

**Figura 7:** Imagem ultrassonográfica da veia safena parva (mesmo caso da Figura 6) com refluxo, demonstrando que o refluxo da veia de Giacomini é transferido para a veia safena parva. VCPC = Veia Circunflexa Posterior de Coxa (Veia de Giacomini); VSP= Veia Safena Parva; JSP= Junção Safenopoplítea.

## DISCUSSÃO

A definição de VG é um pouco controversa na literatura. A descrição original de Carlo Giacomini, de 1873, refere oito tipos diferentes. O tipo 1, adotado em nosso estudo, é o mais comum (52,9%) e descrito como um ramo anastomótico entre a VSP (terminada na veia poplítea) e a VSM^5^ .

A International Union of Phlebology (UIP), apoiada pelo Federative International Committee on Anatomical Terminology, determinou em 2001 uma nova terminologia para as veias superficiais dos MMII, na qual a veia intersafênica corresponde à veia de Giacomini. Da mesma forma, o International Interdisciplinary Consensus Committee on Venous Anatomical Terminology, em 2002, referiu que, quando a extensão cranial da VSP se comunica (via veia circunflexa posterior de coxa) com a VSM, deve ser chamada de VG^6,7^ .

A prevalência de VG varia de 2,5% em estudo flebográfico^8^ , 2 a 86% em avaliações ultrasonográficas^9,10^ e até 95% em dissecação cadavérica^11-13^ .

No nosso estudo, a prevalência baseada na ultrassonografia foi de 14,5% em uma população específica de mulheres encaminhadas para mapeamento venoso para cirurgia de varizes de MMII.

Em relação ao calibre da VG nas 150 veias com refluxo avaliadas em nosso estudo, o diâmetro variou entre 1,5 e 7,8 mm (média de 2,7 mm); com profundidade média de 11,5 mm, variando entre 4 e 25 mm. Delis et al. relataram um calibre médio (2,68 mm) semelhante ao nosso estudo, variando entre 0,2 e 7,7 mm^14^ .

Considerando que a VG apresenta segmentos subfaciais e subcutâneos, acreditamos que a profundidade da VG na presença de refluxo é uma informação relevante no contexto de eventual tratamento endovascular. Tal informação não está disponível na literatura.

Existem seis padrões de refluxo descritos para a VSP incluindo refluxo perijuncional, proximal, segmentar, multissegmentar (com e sem envolvimento da JSP) e distal^15,16^ .

Desses padrões, o perijuncional (refluxo na VSP abaixo da JSP) relaciona-se diretamente com a VG. Nesse padrão, a VG insuficiente transfere refluxo para a VSP abaixo da JSP tornando-a incompetente e com potencial de originar veias varicosas na perna, e, nesse caso, o não tratamento da VG pode gerar recidiva futura.

Na literatura, o refluxo na VG detectado pela USVD varia entre 2 e 19%^17,18^ . Nosso estudo identificou refluxo em 29,4% das VGs, sendo a grande maioria (91,3%) segmentar e somente 8,7% difuso. A maioria do refluxo (66%) é drenada pela JSP, sem causar refluxo na VSP. A maior incidência de refluxo no nosso estudo pode estar relacionada ao fato de a avaliação da VG ser realizada de forma rotineira no laboratório vascular.

Nas VGs incompetentes, 22,7% transferiram refluxo para a VSP, configurando o padrão perijuncional. Veltman et al. encontraram pela USVD 10% de refluxo perijuncional em 1.142 membros inferiores causado por veia de Giacomini ou extensão cranial da VSP^19^ .

Considerando a incidência de refluxo em 29% nas VGs e a possibilidade de padrão de refluxo perijuncional em 23% das VSPs, entendemos que, no mapeamento pré-operatório das varizes dos MMII, deve ser incluída rotineiramente a procura ativa e a avaliação da VG.

Nos casos de refluxo na VG, deve ser mensurado o calibre e a altura das conexões entre a VSM e VSP, que no nosso estudo apresentaram uma altura média (em relação à base do pé) de 71 cm para a VSM e 50,5 cm para a VSP.

Em conclusão, a incidência da VG no estudo foi de 14%. Um terço das veias apresentou refluxo, na maioria das vezes segmentar. Em 23% das VG, ocorre transferência de refluxo para a VSP, fato que torna relevante a sua avaliação durante o mapeamento pré-operatório das varizes dos MMII e para o planejamento cirúrgico.
